# The Role of YY1 in the Regulation of LAG-3 Expression in CD8 T Cells and Immune Evasion in Cancer: Therapeutic Implications

**DOI:** 10.3390/cancers17010019

**Published:** 2024-12-25

**Authors:** Adam Merenstein, Loiy Obeidat, Apostolos Zaravinos, Benjamin Bonavida

**Affiliations:** 1 Department of Microbiology, Immunology & Molecular Genetics, David Geffen School of Medicine, Jonsson Comprehensive Cancer Center, University of California, Los Angeles, CA 90095, USA; adammerenstein@g.ucla.edu; 2 Cancer Genetics, Genomics and Systems Biology Laboratory, Basic and Translational Cancer Research Center (BTCRC), 1516 Nicosia, Cyprus; lo222421@students.euc.ac.cy (L.O.); a.zaravinos@euc.ac.cy (A.Z.); 3 Department of Life Sciences, School of Sciences, European University Cyprus, 1516 Nicosia, Cyprus

**Keywords:** cancer resistance, immune evasion, LAG-3, YY1, CD8 T cell exhaustion, checkpoint inhibitors, immune therapeutics

## Abstract

The treatment of cancers with immunotherapies has yielded significant milestones in recent years. Amongst these immunotherapeutic strategies, the FDA has approved several checkpoint inhibitors (CPIs), primarily Anti-Programmed Death-1 (PD-1) and Programmed Death Ligand-1/2 (PDL-1/2) monoclonal antibodies, in the treatment of various cancers unresponsive to immune therapeutics. Such treatments resulted in significant clinical responses and the prolongation of survival in a subset of patients. However, not all patients responded to CPIs, due to various mechanisms of immune resistance. One such mechanism is that, in addition to PD-1 expression on CD8 T cells, other inhibitory receptors exist, such as Lymphocyte Activation Gene 3 (LAG-3), T cell Immunoglobulin Mucin 3 (TIM3), and T cell immunoreceptor with Ig and ITIM domains (TIGIT). These inhibitory receptors might be active in the presence of the above approved CPIs. Clearly, it is clinically challenging to block all such inhibitory receptors simultaneously using conventional antibodies. To circumvent this difficulty, we sought to target a potential transcription factor that may be involved in the molecular regulation of more than one inhibitory receptor. The transcription factor Yin Yang1 (YY1) was found to regulate the expression of PD-1, LAG-3, and TIM3. Therefore, we hypothesized that targeting YY1 in CD8 T cells should inhibit the expression of these receptors and, thus, prevent the inactivation of the anti-tumor CD8 T cells by these receptors, by corresponding ligands to tumor cells. This strategy should result in the prevention of immune evasion, leading to the inhibition of tumor growth. In addition, this strategy will be particularly effective in a subset of cancer patients who were unresponsive to approved CPIs. In this review, we discuss the regulation of LAG-3 by YY1 as proof of principle for the potential use of targeting YY1 as an alternative therapeutic approach to preventing the immune evasion of cancer. We present findings on the molecular regulations of both YY1 and LAG-3 expressions, the direct regulation of LAG-3 by YY1, the various approaches to targeting YY1 to evade immune evasion, and their clinical challenges. We also present bioinformatic analyses demonstrating the overexpression of LAG-3, YY1, and PD-L1 in various cancers, their associations with immune infiltrates, and the fact that when LAG-3 is hypermethylated in its promoter region it correlates with a better overall survival. Hence, targeting YY1 in CD8 T cells will result in restoring the anti-tumor immune response and tumor regression. Notably, in addition to the beneficial effects of targeting YY1 in CD8 T cells to inhibit the expression of inhibitory receptors, we also suggest targeting YY1 overexpressed in the tumor cells, which will also inhibit PD-L1 expression and other YY1-associated pro-tumorigenic activities.

## Simple Summary

Several cancer treatments that have been used for quite a few decades include surgery, chemotherapy, and radiation. These resulted in significant responses and the prolongation of survival for a subset of cancer patients. Another subset was not responsive to such treatments and many of the originally responding patients became unresponsive to subsequent treatments and succumbed to the disease. Fortunately, a new treatment was introduced that takes advantage of the patient’s immune response to fight the cancer and eradicate it, as it does with viral and microbial infections too. The immune response to cancer is called “immunotherapy”. Several immunotherapeutic approaches were developed and approved by the FDA for various cancers, and yield significant clinical responses and the prolongation of survival in many cancer patients. Immunotherapy depends largely on the patient’s immune response being activated and killing the tumor cells. Killing the tumor cells is mediated by immune cells, called CD8 T cells, which need to locate and bind to the cancer cells to kill them. Interestingly, cancer cells strive to survive and escape their death via the CD8 T cells. They manifest this clever approach by inducing various markers on their surface that will prevent them from killing the cancer cells, resulting in the survival and growth of the cancer cells. Therefore, such a failure of immunotherapy required a remedy. Indeed, a remedy was found through which these markers were inhibited therapeutically by agents approved by the FDA, resulting in the cancer cells’ ability to inhibit the CD8 T cells being overridden. Such approved agents were very effective in many cancer patients, but unfortunately not all. Here too we needed to develop other therapies to treat the unresponsive patients. Here, we describe a novel approach that involves using an agent that inhibits the expression of the above inhibitory markers on the CD8 T cells, allowing them to be active and kill the cancer cells in unresponsive patients. It is worthwhile to develop this new approach through research investigation and clinical trials.

## 1. Introduction

Yin Yang 1 (YY1) is a zinc-finger transcription factor that regulates the activation and repression of transcription [1]. YY1 repression or activation has been linked to gene dependence. YY1 has been known to interact with DNA-binding cofactors that can recruit promoters to increase transcriptional activity [2,3]. The zinc-finger binding domain of YY1 overlaps with parts of the transcriptional repression sequences, which leads to the repression of transcriptional activity [4]. The YY1 activation transcription factor 6 (YY1-AFT6) interacts with the gastrin-releasing peptide (*GRP*) genes to increase its transcription [1,5]. YY1 also interacts with the inositol-requiring mutant 80 (INO80) to bring in promoter sites that influence cell division, DNA replication, genomic stability, and DNA repair [1,6]. In addition, YY1 affects the repression or activation of different transcriptional activities throughout the cell [1].

YY1 interacts with the G1/S phase transition. This has been shown through its change in localization sequences during this transition. At the beginning of the S phase, YY1 shows subcellular nuclear localization. During the second half of the S phase, YY1 shows subcellular localization to the cytoplasm. This change in subcellular localization confirms that YY1 interacts with the G1/S phase [1,7]. The YY1 regulation of the expression of genes influences development, cell proliferation, differentiation, DNA repair, and apoptosis. YY1 interacts with and modifies the chromatin’s 3D organization to regulate these different genes [8]. YY1 chromatin regulation affects various tumor signaling pathways, including c-myc, c-fos, HER2, E1A, and p53 [8,9,10]. The YY1 regulation of certain genes also plays a role in sustained proliferative signaling, evading programmed cell death, and deregulated metabolism [8]. YY1 is upregulated in various cancers, including breast, bladder, cervical, colon, esophageal, liver, brain, and gastric cancers [11]. The overexpression of YY1 has been further linked to tumor cell resistance in cell-mediated immunotherapies [3]. Research investigation on YY1 has shown its importance in various cancer pathways, and many different possible treatment methods. Furthermore, it is possible to find cancer subtypes in which YY1 overexpression is associated with cancer progression, and other subtypes in which its overexpression has a tumor-suppressive effect. Accordingly, YY1 prognostic behavior changes according to the tumor type [12].

The lymphocyte activation gene 3 (LAG-3) is located on chromosome 12 in humans and encodes a 498 amino acid protein [13,14,15]. LAG-3 is necessary for T cell regulation and homeostasis within the immune system [15]. LAG-3 is an inhibitory receptor on T cells that causes their inactivation when it binds to its respective ligand. There are multiple different ligands that have been shown to bind to LAG-3, namely, MHC Class II, Galectin-3, Fibrinogen-like protein 1 (FGL-1), and Liver Sinusoidal Endothelial Cell Lectin (LSECtin) [14,15]. LAG-3 is involved in the downregulation of the expression of CD3^+^, CD4^+^, and CD8^+^ T cells by binding to MHC class II molecules on antigen-presenting cells, effectively suppressing the activation of immune response and downregulating these T cells [16]. When LAG-3 is inhibited, it can lead to a stronger T cell response to certain tissues like cancer. LAG-3 has been shown to be an important inhibitory pathway involved in tumor development, along with other inhibitory checkpoints [17,18,19]. Donia et al. [19] reported how MHC Class II can be constitutively expressed by interferon gamma (IFNγ). In the study, they also demonstrated how the increased expression of MHC Class II is a strong stimulus that promotes the accumulation of tumor-specific CD4^+^ T cells in the tumor microenvironment. Furthermore, they showed how TNF from CD4^+^ T cells decreases CD8^+^ T cell reactivity. Thus, increased CD4^+^ recruitment from the MHC-class II expression has been linked to lower CD8^+^ T cell reactivity [14,18]. Galectin-3, LSECtin, and FG-1 ligands binding to LAG-3 regulate T cell activation and function. The interaction of LAG-3 with these three ligands inhibits the proliferation and cytotoxicity of NK/CTL cells within the immune response [16,17,20,21,22]. LAG-3 inhibitors have been shown to increase CD8 T cell activation and proliferation, and this inhibition has led to an increase in immune response against tumors [23].

YY1 is involved in immune suppression and the inhibition of CD8 T cell function, in part via the upregulation of checkpoint inhibitors (CPIs) [24]. Likewise, the expression of LAG-3 on CD8 T cells is also involved in the inactivation and the exhaustion of CD8 T cells [16].

Clearly, due to the failure of responses to CPIs in a subset of cancer patients and not all cancer types responding to CPIs, a novel approach is needed to prevent immune evasion and CD8 T cell exhaustion. We hypothesized that the inhibition of LAG-3 expression, by inhibiting its regulation by YY1, offers an alternative to CPIs that could lead to the restoration of the anti-tumor CD8 T cell function and be effective in cases of resistance to CPIs. Hence, we provide a new approach, whereby inhibiting the expression of inhibitory receptors on CD8 T cells, rather than blocking the receptors, circumvents the immune evasion and restores the anti-tumor response. Such a novel approach is addressed in this report. Herein we discuss the role of YY1 as a transcription factor in the regulation of CD8 T cell function and immune evasion, the significance of LAG-3 and YY1 expressions on anti-tumor CD8 T cells and immune evasion, the molecular regulations of the expression of LAG-3 on CD8 T cells, the mechanisms by which YY1 regulates the expression of LAG-3, bioinformatic analyses on the YY1-LAG-3 axis in various cancers, approaches to therapeutically targeting YY1, and future directions and challenges for clinical therapeutic applications.

## 2. YY1 as a Transcription Factor

### 2.1. Overview of YY1’s Structure and Function

**Figure 1:**
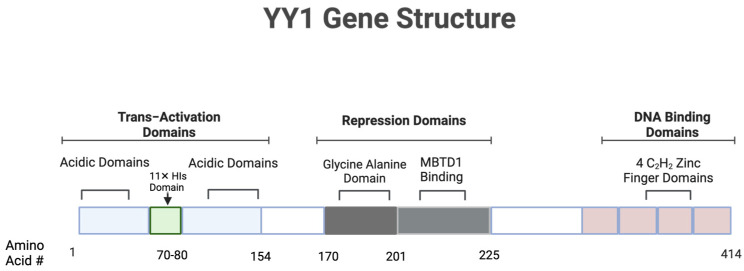
YY1 gene structure. This figure provides a comprehensive depiction of the Yin Yang 1 (YY1) transcription factor, illustrating its key structural domains and functional regions. YY1 is 414 amino acids long and consists of three major domains. The transactivation domains have acidic domains of around 70 amino acids each. The histidine domains for activation of YY1 have 11 histidines in a row. The repression domains have a 32 amino acid-long Glycine Alanine Domain and a 25 amino acid-long MBTD1-binding domain. Finally, in the DNA-binding domains of YY1, there are four zinc finger domains. YY1 contributes to cancer progression and immune evasion in various ways. YY1 regulates the protein stability and expression of many different cancer-associated genes. YY1 also contributes to the upregulation or downregulation of various T cell stability and regulation processes, contributing to a much stronger immune evasion response. Prepared by BioRender, Inc. (Toronto, ON, Canada).

YY1 is a transcription factor that regulates the activation and repression of transcription [1,24]. YY1 can function as an activator, coactivator, repressor, or initiator of transcription [25]. YY1 is thought to have transcriptional control of approximately 10% of the entire mammalian gene set [26,27]. YY1 in humans consists of 414 amino acids. YY1 has a transactivation domain, repression domain, and 4${\;4\;C}_{2}H_{2}$- type zinc fingers [27]. From residues 295–414, the crystal structure showed the $C_{2}H_{2}$ zinc finger fold (see Figure 1). Another region, from residues 206–225, when in interaction with the Malignant Brain Tumor domain containing 1 (MBTD1) protein from Drosophila, assumes a conformation of two anti-parallel β–strands connected by a β-hairpin loop [27,28]. Furthermore, there are multiple amino acid sequence motifs that reside within the YY1 N-terminus. Residues 16–29 form an amphipathic acidic helix, residues 43–53 form a stretch of 11 consecutive aspartic and glutamic acids, residues 54–69 form a glycine-rich segment, residues 70–80 form a stretch of 11 consecutive histidine residues, 80–100 form a region enriched with glutamine and proline, and finally, residues 154–201 form a segment enriched with alanine and glycine [27,29,30]. However, more research is needed to understand the role that each of these different amino acid motifs play in the function of YY1’s role in its different pleiotropic anti-tumor activities.

Through a wide range of studies, Weintraub et al. [8] provided evidence that YY1 contributes to the enhancer–promoter structural interactions. YY1 binds to these enhancer–promoter regions and was shown to be required for normal levels of gene transcription. Weintraub et al. [8] further showed that YY1-mediated enhancer–promoter interactions were necessary for adult stem cell and embryonic viabilities, showing the importance of YY1 within the body [8]. Not only does YY1 act as a transcription factor, but also as a transcription cofactor. YY1 enhances the association of androgen receptors with the androgen response element. It does not directly act on the transcription process, but instead acts as a cofactor to stimulate the effects of the androgen receptor to a great extent [31,32]. This is just one pathway in which YY1 can act as a transcriptional cofactor. Not only can YY1 act as a transcriptional cofactor, but it can also act as a cofactor for other purposes. In a study by Donohue et al. [33], they showed that YY1 acts a cofactor and interacts with the CCCTC-binding factor (Ctcf) and X-inactive specific transcript (XIST) antisense RNA (Tsix). This interaction was shown to cause different fates for the X chromosome in females. Chromatin immunoprecipitation (ChIP) was used to determine when the YY1–Tsix interactions occurred at different differentiation stages in mouse embryos. There was consistent binding at the X chromosome in both XY and XX cells of this interaction. This Ctcf-YY1-Tsix axis showed that YY1 as a cofactor was key for the X chromosome binary switch [33].

### 2.2. YY1’s Role in Gene Regulation

The epithelial–mesenchymal transition (EMT) is the process by which epithelial cells lose their cell–cell adhesion and polarity to become migratory mesenchymal stem cells (MSCs) [34,35,36]. There has been a clear link between EMT and metastasis and growth in cancer. YY1 is key to understanding the progression of EMT in cancer. YY1’s participation has been established in the nuclear factor kappa beta (NF-κB)/Snail/YY1/Raf-1 kinase inhibitory protein (RKIP) circuitry loop (NF-κB)/YY1/SNAIL/RKIP) that regulates EMT [36,37,38]. YY1 promotes EMT through the NF-κB/Snail/YY1/RKIP loop via its transcriptional upregulation of the zinc finger protein Snail, which was found to induce EMT [34,36,37,38]. The upregulation of Snail induces EMT through the repression of epithelial markers such as E-cadherin and claudins. These two factors promote anti-invasive and anti-migratory properties in epithelial cells [36,39].

YY1 has been shown to both negatively and positively regulate transcriptions of certain genes. For the negative regulation, there are three different methods that have been suggested. First, YY1 hinders the binding of transactivators to DNA sterically, by binding competitively to promoter elements with overlapping DNA recognition sites for transcriptional activators. The second proposed mechanism is that YY1 inhibits the interaction of activators with the general transcriptional machinery or masks the general factors from an activator. Finally, the third proposed method is that YY1 recruits a co-repressor or complex to a promoter, which then negatively affects other factors that are present or alters the local chromatin structure for repression [39]. The most likely outcome for the repression of the transcription of many YY1-regulated genes is that it uses a combination of these factors altogether to repress the transcription. Furthermore, the positive regulation of many different YY1-targeted genes has multiple different pathways for different genes as well. For example, YY1 directly interacts with general transcription factors like the TATA-binding protein (TBP), Transcription initiation factor subunit 55 (TAFII55), Transcription factor II B (TFIIB), and Androgen Receptor (AR) to activate transcription [31,40]. YY1 can also unmask activator domains or inhibit repressor activity [31]. YY1 also interacts with CBP and p300, which are both histone acetyltransferases that can change chromatin structure and cause an increase in the transcriptional activity of the genes these cofactors bind to [40,41]. YY1 plays a very vital role in either upregulating or downregulating genes, in the ways that are described above. For example, YY1 has been shown to recruit co-repressors such as the Smad family members, which then, in turn, repress TGF-β signaling and cell differentiation [4,42]. Furthermore, in mammary epithelial cells, YY1 represses β-casein by outcompeting the mammary gland specific factor (MGF) of the STAT family, STAT5A [4,43]. The YY1 binding site is the same as the activator site on the DNA; therefore, it then can bind to the activator site, and outcompetes the β-casein. Also, Wu and Donahue [44] reported that in mouse neurons, YY1 and its interacting partner Bromodomain containing protein 4 (Brd4) activate sentrin-specific protease 1 (Senp1). Senp1 is an upstream regulator of glutamate signaling, which plays a pivotal role in neuronal plasticity. YY1 has a clear importance in the control of gene expression throughout mammalian cells [44].

### 2.3. YY1 in Cancer

The manipulation of YY1 expression in different cancer cell lines has been shown to have both negative and positive outcomes on the prevalence of cancer. For example, YY1 knockdown completely abolishes the tumor-forming ability of the high tumorigenic bladder cancer cell line UMUC3 [40,45]. Furthermore, YY1 depletion has also been shown to stop growth in soft agar in vitro of bladder, prostate, lung, and breast cancer cell lines [40]. YY1 regulates the protein stability and expression of many different cancer-associated genes as well [40,46]. YY1 promotes P27 ubiquitination and physically interacts with P27 in various breast cancer cell lines [40,46]. P27 is almost always degraded in cancer cell lines, showing how YY1 can increase the likelihood of breast cancer through this ubiquitination. Furthermore, YY1 binds to the BRCA1 promoter and increases its expression of BRCA1 and other downstream genes in breast cancer [40,47]. YY1 interacts physically with the suppressor of zeste 12 (SUZ12) and recruits certain polycomb proteins and DNA methyltransferases to participate in gene silencing of the tumor suppressor CEBPD [40,48]. Knocking down YY1 inhibits, whereas overexpression activates the Wnt/β-catenin pathway in gastric adenocarcinoma (GAC) [40,49]. YY1 can promote tumor progression and survival by activating certain oncogenes such as c-Myc and cyclin D1, and surviving [11,44]. Hence, YY1 has countless different roles in both the upregulation of certain oncogenes and the suppressing of different tumor suppressor genes [50].

## 3. Molecular Regulation of LAG-3 Expression

### 3.1. General Properties of LAG-3

The lymphocyte activation gene 3 (*LAG-3*) is located on chromosome 12 in humans and encodes a 498 amino acid protein [14,15]. LAG-3 is a cell surface molecule that is expressed on activated T cells, Natural Killer (NK) cells, B cells, and plasmacytoid dendritic cells [12,51]. LAG-3 is an inhibitory receptor on T cells that causes the inactivation of T cells when the ligand binds to LAG-3. The ligands that have been reported to bind to LAG-3 are MHC Class II, Galectin-3, Fibrinogen-like protein 1 (FGL-1), and Liver Sinusoidal Endothelial Cell Lectin (LSECtin) [12,14].

**Figure 2:**
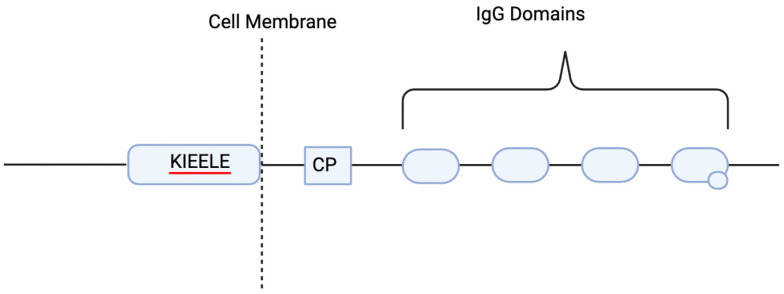
The KIELLE domain in LAG-3. This figure depicts the structural organization of the LAG-3 protein, a key inhibitory receptor involved in the regulation of immune responses. The KIELLE domain, the CP domain, and the IgG domains. Created by BioRender, Inc.

**Figure 3:**
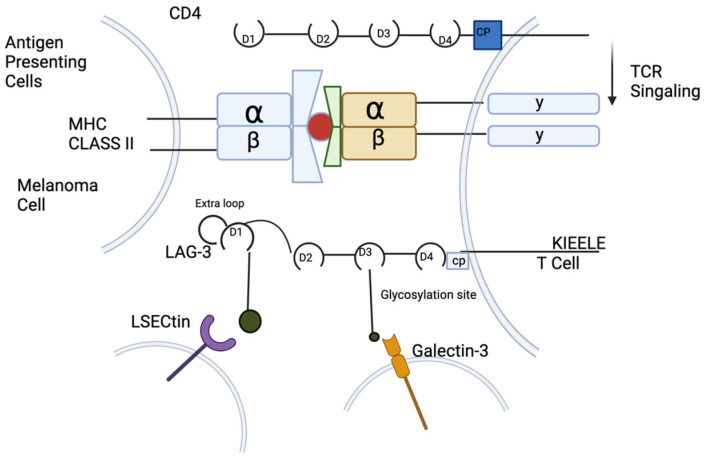
LAG-3 higher affinity for MHC-II. CD4 cells use four extracellular immunoglobulin superfamily-like domains (d1–d4). LAG-3 utilizes the extra loop with 30 amino acids in D1 to bind to MHC class II with greater affinity. Ligation of MHC class II, by antigen presenting cells or aberrantly by melanoma cells, with LAG-3 mediates an intrinsic negative inhibitory signal, in which the KIEELE motif in the cytoplasmic domain is indispensable. LAG-3 is highly glycosylated with LSECtin, expressed on melanoma cells, and Galectin-3 is expressed on stromal cells and CD8^+^ T cells in the tumor microenvironment. This figure shows the interaction between LAG-3 and these three ligands and how it interacts with CD4 and CD8 T cells. Created by BioRender, Inc.

LAG-3 is a transmembrane protein that includes four extracellular IgG domains that have strong structural homology to CD4. The intracellular domain of LAG-3 contains a unique amino acid sequence (KIEELE) that is required for LAG-3 to be able to have a negative effect on T cell function [52] (see Figure 2). In a study by Workman and Vignali (2003), they reported that LAG-3-expressing T cells without the KIELLE domain could not negatively influence T cell function, like what was observed when LAG-3 was present with the KIELLE domain [53]. However, the importance of this motif has been contested by others. For example, one study found that deletion of the KIEELE motif had no impact on LAG-3 function [54]. However, when KIELLE mutations combined with what was called the FxxL motif mutations showed changes in immune response, pairing the mutations within the FxxL with the deletions of part of the KIEELE motif nearly doubled IL-2 production, which surprisingly turned LAG-3 into a positive co-receptor [54]. LAG-3 binding capabilities being changed can have a negative regulatory role for the LAG-3/Class II MHC interaction. Using a series of mixed lymphocyte reactions, soluble LAG-3 clearly down-modulated human CD4 T cell function in vitro. LAG-3 binds to MHC class II molecules, but with a much higher affinity than CD4 T cells can, which shows that LAG-3 might act as a negative competitor of CD4, which can lead to down-modulated CD4 [53] (Figure 3).

The cytoplasmic tail of LAG-3 plays a critical role in the function of LAG-3, as its deletion completely abrogates its inhibitory function. However, the mechanism by which the cytoplasmic tail mediates this is still uncertain, and being researched now [55]. For LAG-3 to be expressed on the cell surface, T cell activation is a necessary requirement [55,56]. Bae et al. [56] examined the trafficking of human LAG-3 in both unstimulated and stimulated conditions of T cells. During its unstimulated condition, the majority of LAG-3 did not reach the cell surface and was degraded in lysosomal compartments. During its T cell stimulation condition, the majority of LAG-3 translocated to the cell surface without degradation in the lysosomal compartments [56].

PD-1 and LAG-3 are both inhibitory receptors. PD-1 and LAG-3 have been shown to be similar and generally co-expressed in cancer [11]. In their study, Jiang et al. [57] showed that the combined targeting of both LAG-3 and PD-1 may lead to the prolonged inhibition of immunosuppressive pathways. This response was seen to be better than just targeting one or the other, thus showing the likelihood of the similarity and co-expressions of PD-1 and LAG-3 in cancer cells. Jiang et al. [57] created a bispecific antibody called IBI323 that was shown to inhibit both PD-1 and LAG-3. They evaluated the in vivo activity of IBI323 in PD-L1/LAG-3 double knock-out in a mouse model bearing human PD-L1 knock-in MC38 colon adenocarcinoma. Their results showed that IBI323 significantly inhibited MC38 tumor growth through both CD8^+^ and CD4^+^ T cells and led to an increased number of cytokines producing cancer-specific T cells in the tumors and blood [57].

### 3.2. Transcriptional Regulation of LAG-3

Transcription of the *LAG-3* gene produces four transcripts or splice variants (LAG-3-201, LAG-3-202, LAG-3-203, and LAG-3-204). At the transcriptional level, multiple transcription regulators have been found to regulate the expression of LAG-3. These include the thymocyte selection-associated high mobility group box protein (TOX) [58], a nuclear factor of activated T cells family member [59], the nuclear receptor subfamily 4 group A (NR4A1) [23,60], and the early growth response gene 2 (EGR2). LAG-3 has been shown to be a gene target for EGR2. The expression of Egr2 and LAG-3 helped illuminate a global state of T cell differentiation associated with T cell dysfunction in vivo [61]. Also, the transcription of LAG-3 was found to be reduced by glycogen synthase kinase-3 (GSK-3). Rudd et al. [62] showed that GSK-3 was more effective than a LAG-3 blockade alone in suppressing B16 melanoma growth. This occurred through an increase in the expression of the transcription factor Tbet by GSK-3. Tbet binds to the LAG-3 promoter, which in turn inhibits the transcription of LAG-3, causing the decrease in LAG-3 transcription [61,62].

### 3.3. Epigenetic Regulation of LAG-3

CpG islands located in the LAG-3 promoter region were shown to be highly methylated, further providing evidence that LAG-3 is likely regulated by DNA methylation [55]. The epigenetic regulation of LAG-3 and its different transcripts are still relatively unknown; however, there is belief that it is most likely regulated through methylation. This is because genes that have been shown to have similar functions to LAG-3, like PD-L1 and CTLA-4, have all been proven to be epigenetically modified by DNA methylation [55,63].

### 3.4. Post-Transcriptional Regulation of LAG-3

Many miRNAs have been shown to regulate LAG-3. miRNAs are small non-coding RNAs that carry out post-transcriptional regulation of gene expression. MicroRNAs have been shown to regulate gene expression by blocking mRNA translation [64]. In head and neck squamous cell carcinoma (HNSCC), microRNA (miR)-7704 and miR-21-5p were shown to have the capability to increase the expression of LAG-3 [61]. Zhao et al. [61] performed a study to track the effects of these miRNAs on LAG-3. They showed that transfection of miR-7704 or miR-21-5p significantly elevated LAG-3 mRNA levels compared to the control. However, the method that these miRNAs use in the transcriptional regulation of LAG-3 is still unknown and needs to be further researched [61]. Many other miRNAs have been shown to regulate inhibitory receptors like LAG-3. For example, more than 50 miRNAs regulate the PD-1/PD-L1 pathway [63]. However, there are not as many data on the LAG-3-specific miRNAs, other than microRNA (miR)-7704, miR-21-5p, and miR-16. However, the mechanism has not yet been studied, and further research is needed into understanding how this regulation occurs. Also, miR-16 regulates the PD-1/PD-L1 pathway too [64]. N6-methyladenosine (m6A) modifications are also involved in the post-transcriptional regulation of LAG-3. For example, the AlkB homolog 5 (ALKBH5) and YTH domain family member 1 (YTHDF1), which act as RNA demethylase during m6A modifications, have been associated with the expression of LAG-3 [59,63]. In addition, checkpoint expression levels are higher in colon cancer patients with higher levels of ALKBH5 and lower levels of YTHDF1 expression [61,64].

### 3.5. Translational Regulation of LAG-3

Post-translationally, LAG-3 is translocated to the cell surface upon stimulation, through protein kinase C signaling in an intracellular domain-dependent manner [65,66]. However, LAG-3 is degraded in lysosomes in the absence of antigen stimulation, but the specific mechanism of translation remains to be determined [61,65,66,67]. LAG-3 cleavage by A Disintegrin and metalloproteinase domain-containing protein 10 (ADAM10) and ADAM17 produce a soluble version of the protein (sLAG-3), which alleviates T cell inhibition, positively regulates CD8^+^ T cells, interleukin (IL)-12, IFN-γ, and dendritic cells (DCs), and functions as a prognostic marker in multiple cancers [61,65,66,67]. Furthermore, LAG-3 cytoplasmic tailless mutants lack the ability to compete with CD4. They cannot sustain the inhibitory effects of LAG-3. This suggests that the transmission of an inhibitory signal via the LAG-3 cytoplasmic domain is a critical aspect of its function [68,69]. The LAG-3 cytoplasmic domain has an unusual motif consisting of glutamic acid and proline di-peptide repeats (“EP motif”) [69]. The EP motif regulates the magnitude of TCR-induced signaling through the dissociation of tyrosine kinase Lck from CD4 or CD8 co-receptors [69].

## 4. YY1-Mediated Regulation of LAG-3 Expression

YY1 has been shown to regulate the expression of LAG-3 and similar inhibitory checkpoints like PD-1 as well. It regulates both checkpoints by binding to their promoters [70]. The upregulation of PD-1 and LAG-3 results in negative feedback for cytotoxic T cells. Kwiatkowksa et al. [70] reported that human melanoma sections stained by immunofluorescence showed activation of the p38MAPK/JNK pathway in tumor-infiltrating lymphocytes. This pathway drives YY1 expression, leading to PD-1 upregulation and the persistence of melanoma. This means that YY1 has a positive regulation on PD-1 expression. Furthermore, the downregulation of YY1 showed a decrease in the transcription of LAG-3 and PD-1 [70,71]. The downregulation of LAG-3 and PD-1 increases the response against tumors, showing the importance of YY1 in the regulation of these genes [72]. There has been much less research on solely LAG-3-mediated expression by YY1, and this mechanism will be further analyzed below.

Balkhi et al. [71] reported that YY1 also regulates another checkpoint inhibitor (Tim3) and negatively regulates type I cytokines interleukin-2 (IL-2) (in collaboration with Ezh2 histone methyltransferase) and interferon gamma (IFN-γ). Further building on YY1 regulation of PD-1 and LAG-3, they showed that while searching the TRANSFAC database (Biobase), PD-1 and LAG-3 promoters were found to contain consensus binding sites for YY1. Balkhi et al. [71] used gel shift assay to confirm that YY1 binds specifically to the consensus sites present in PD-1 and LAG-3. Reporter gene assays showed increased transcription with repeat T cell stimulations, which was abrogated by mutation of the YY1-binding sites. This confirmed that PD-1 and LAG-3 genes are positively regulated by YY1. The knockdown of YY1 in repeatedly stimulated cells markedly reduced LAG-3, showing further support of positive regulation [71]. Furthermore, Balkhi et al. [71] discovered that components of both the TCR signal 1 and the CD28 signal 2 merge into the p38MAPK/JNK pathway, leading to phosphorylation of cJun and ATF2 and their conversion into their transcriptionally active forms. Phospho-cJun/ATF2 dimers bind to the CRE locus in the YY1 promoter, activating transcription for YY1 and subsequently upregulating LAG-3 expression by binding to its promoter, showing a pathway of LAG-3 expression through YY1 activation. Continuing along this pathway, MAPK inhibition using EGFR and MEK inhibitors prevented expression of PD-L1 at the transcriptional and translational levels in non-small cell lung cancer (NSCLC) [73]. This finding suggested a more marked connection between the MAPK pathway and LAG-3, PD-L1, and YY1. Medhi and Rabbani clearly state the role of communication amongst the exhausted T cells, LAG-3, and YY1. They explain that exhausted T cells have high surface expression of inhibitory molecules like PD-1, TIM3, LAG-3, TIGIT, and 2B4, and that the transcription factors associated with high PD-1 expression are T-bet, Eomes, and YY1 [74,75,76,77]. YY1 can regulate the inhibitory molecules’ PD-1, LAG-3, and TIM3 expressions, and downregulate IL-2 via EZH2 activation, features characteristic of exhausted T cells [74,75,76,77]. The transcription factor YY1 is overexpressed in most tumors and participates in regulating the resistance of tumor cells to cellular immunotherapy. There are several signal crosstalk pathways between YY1 and the regulation of PD-L1 expression, including p53, miR34a, STAT3, NF-kB, PI3K/AKT/mTOR, c-Myc, and COX-2 [3,78,79,80]. Therefore, the role of YY1 in tumor immune resistance may be related to the overexpression of PD-L1 on cancer cells and plausibly LAG-3 on CD8 T cells. Another pathway that LAG-3 and YY1 may interact through is the expression of FOXP3. YY1 can block the expression and function of Foxp3, which in turn blocks the development of Treg cells [81,82]. Foxp3 is an important molecule in the differentiation of Treg cells [82]. Like YY1, alluding to possible crosstalk, LAG-3 was also shown to inhibit the cytotoxic function and proliferation of Treg cells like YY1 in a separate study.

## 5. Roles YY1 and LAG-3 in Immune Evasion

### 5.1. Role of YY1 in Immune Evasion

YY1 contributes to immune evasion in various ways. For example, one pathway by which YY1 can lead to immune evasion is by upregulating certain genes that interact with Foxp3. Foxp3 is involved in the control of differentiation and the function of regulatory T cells (T_reg_). T_reg_ cells play a critical role in maintaining immune homeostasis. T_reg_ cells inhibit differentiation and the proliferation of conventional T (T_conv_) cells, including Th1, Th2, Th17, and Tfh cells. T_reg_ cells help prevent excessive immune responses against self-antigens, food antigens, commensal microorganisms, and cancers [81,83,84,85]. In their studies, Hwang et al. [81], show that Foxp3 is indispensable for the differentiation and function of T_reg_ cells, specifying the T_reg_ cell lineage. Hwang et al. [81] also showed that when YY1 was introduced into naïve murine CD4 T cells, they were subjected to T_reg_ differentiation conditions. Foxp3 expression was markedly decreased in YY1-transduced T_reg_ cells compared with those in control vector-transduced T_reg_ cells. This showed that YY1 decreases the expression of Foxp3, which stops the body from having the appropriate immune response and T_reg_ cell differentiation. YY1 physically interacts with Foxp3 to directly switch on Foxp3 target genes and interfere with Foxp3-dependent target gene expression. Thus, YY1 inhibits the Treg cell’s differentiation and function by blocking Foxp3 [81,86]. Not only can YY1 inhibit T cell differentiation, it can also stop the activation of already developed T cells within the body. YY1 inhibits the activation of T cells and promotes immune tolerance in tumor cells by activating the expression of inhibitory receptors such as PD-1 [3,11,87]. YY1 further promotes immune evasion through the dysregulated NF-κB/Snail/YY1/RKIP loop [88]. The overexpression of NF-κB, Snail, and YY1 has led to the maintained downregulation of Raf kinase inhibitor protein (RKIP) [89]. This combination led to cell resistance and insensitivity to both chemo and immune-therapeutic drugs [89,90]. RKIP upregulation in tumor cells also resulted in the upregulation of Fas and DR5 via the RKIP-mediated inhibition of NF-κB and YY1 [91].

**Figure 4:**
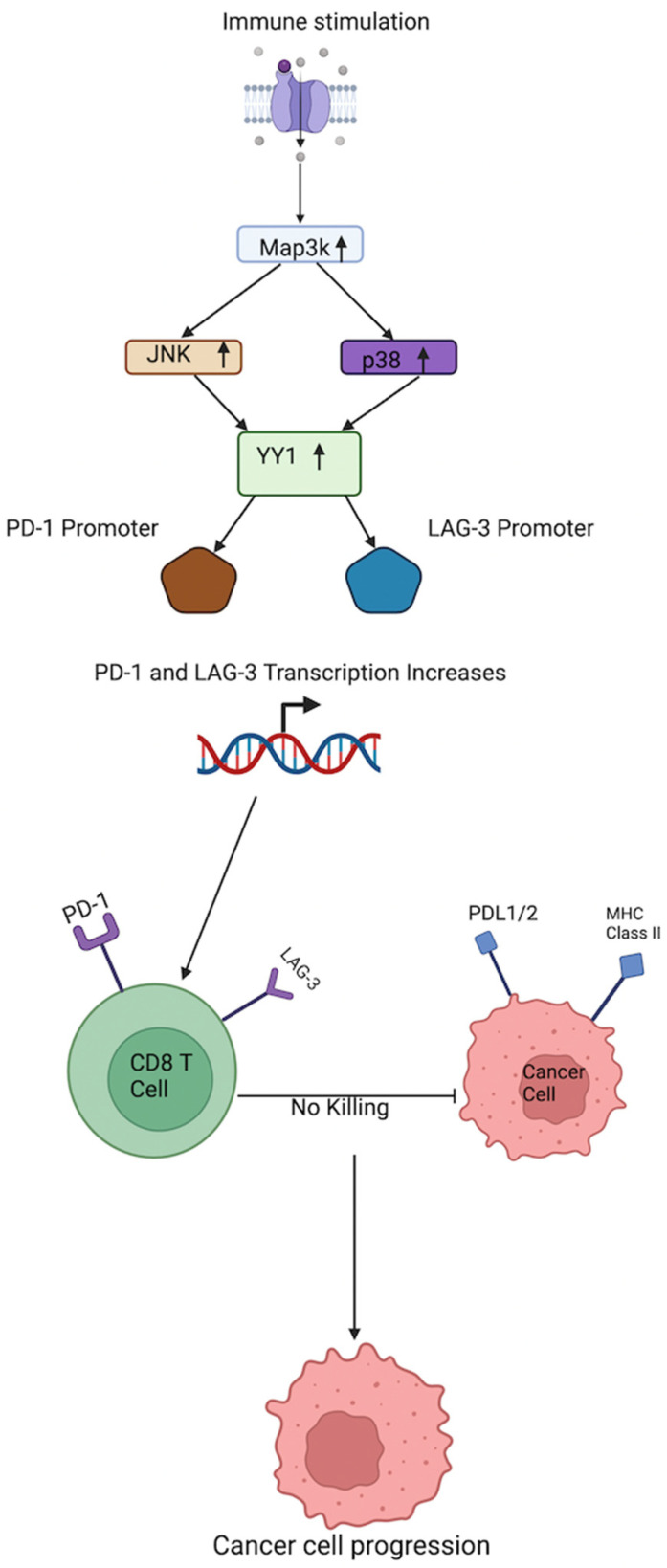
Regulation of PD-1 and LAG-3 by YY1 in tumor-infiltrating CD8 T lymphocytes. This figure depicts p38MAPK/JNK/YY1/LAG-3-PD-1 pathway in tumor-infiltrating lymphocytes. MAP3K activation increases JNK and p38, leading to an increase in YY1 expression. This pathway, which drives YY1 expression, leads to YY1-mediated transcriptional PD-1 and LAG-3 upregulations. The anti-tumor CD8 T cells, expressing both PD-1 and LAG-3 inhibitory receptors, will bind the tumor target cells, leading to the inactivation of the CD8 T cells through their interactions with the PDL-1/2 and MHC-II, respectively. Thus, tumors escape via immune evasion and tumor growth. Created by BioRender, Inc.

In immunotherapy, T cell exhaustion is a phenomenon that affects CD8^+^ T cells, where prolonged antigen exposure renders the cells hyporesponsive and incapable of eliminating tumor cells. T cells normally assist in eradicating malignancy or infections; however, dysfunctional T cells, which lead to exhaustion, may cause resistance and the survival of tumors [1,71,92]. Balkhi et al. [71] described that in T cell exhaustion, cytokine deprivation and checkpoint receptor action are critically important, and that YY1 can regulate these elements. YY1 positively regulates the PD-1 [71,93], Tim3 [71,93], and LAG-3 [71,93] checkpoint inhibitors, but negatively regulates IL-2 [93,94] and IFN-γ [93,94], both of which are type I cytokines [71,93,94]. As a type I cytokine, IL-2 plays a pivotal role in clonal expansion and the persistence of virus and tumor-reactive T cells [71,93,94]. YY1 has been shown to have similar binding sites to the TATA binding protein in IL-2, which is the method it uses to have gene repression on IL-2. Furthermore, there are other epigenetic changes that YY1 makes to further repress IL-2. In the study, Balkhi et al. [71] showed that after YY1 repressed the Il-2 gene activation, repeated in vitro stimulation led to CD8 and CD4 T cells showing markers of PD-1, LAG-3, and Tim3, which are all signs of T cell exhaustion. Furthermore, Balkhi et al. [71] also showed that PD-1 and LAG-3 were indeed positively regulated by YY1. A gel shift assay confirmed that YY1 binds specifically to the consensus sites present in PD-1 and the LAG-3 promoters. Reporter gene assays showed increased transcription with repeat T cell stimulations and less increase in transcription by mutation of the YY1-binding sites, confirming that PD-1 and LAG-3 genes are positively regulated by YY1 [71]. High levels of PD-1 have been shown to cause exhaustion of tumor-infiltrating lymphocytes and the inability to have a correct immune response in the presence of cancer [71]. In a study by Kwiatkowska et al. [70], they stained human melanoma sections by immunofluorescence, and these showed activation of the p38MAPK/JNK pathway in tumor-infiltrating lymphocytes, which drives YY1 expression, leading to PD-1 and LAG-3 upregulations. MAP3K activation increases JNK and p38. This leads directly to an increase in YY1 expression, which in turn upregulates PD-1 and LAG-3 (70). This process, which ends in the upregulation of PD-1 and LAG-3, is clearly regulated by YY1, and causes T cell exhaustion and the persistence of various cancers (see Figure 4).

Stimulation of PD-1 by PD-L1 or PD-L2 induces the inhibition of T cell proliferation and T cell activity. Consequently, overexpression of PD-L1 in tumor cells can lead to PD-1 activation and the suppression of T cell immune response within the TME [95]. PD-L1 is regulated by YY1 in cancer, where overexpressed PD-L1 by tumor cells hijacks this immune regulatory system, preventing proper targeting and response against cancer cells [96,97].

Balkhi et al. [71] reported that in melanoma, functional exhaustion with increased fractions of PD-1+ CD4 cells was found to be associated with a high level of YY1 [71]. This provides a foundation for a possible YY1- LAG-3 immune exhaustion response. Furthermore, Touboul and Bonavida [98] speculated that targeting YY1 may be both inhibiting PD-1 expression on CD8^+^ CTLs and restoring their anti-tumor activities, and on the other hand, targeting YY1 may be inhibiting PD-L1 on tumor cells, as well as inhibiting tumor cell survival, metastasis, and resistance. They also infer that targeting YY1 may also inhibit LAG-3 and contribute to the various anti-tumor activities outlined above [98]. This inference seems plausible since YY1 was shown to have high mRNA expression in most different types of tumor tissues [86]. Furthermore, YY1 has been shown to both promote and inhibit angiogenesis in different types of cancers [81]. YY1 regulates the general processes of many different stages of T cell and B cell differentiation. YY1 was upregulated in PD-1-positive T cell-infiltrating lymphocytes in melanoma tumors and directly regulated the expression of PD-1 and LAG-3 by binding to the promoter region [86]. Hwang et al. [81] showed that YY1 inhibits the Treg cells’ differentiation and function by blocking Foxp3. YY1 binds to the homologous site of endogenous COX-2 promoter YY1 and enhances its transcriptional activity in macrophages. They showed that YY1 is clearly critical for the tumor-immune microenvironment. YY1 further plays a role in glucose, glutamine, and lipid metabolism, by regulating the transcriptional activation and expression of some molecules in these processes [81,86]. YY1 is involved in tumor resistance to immune checkpoint inhibitors (ICIs) immunotherapy, achieved through the positive regulation of PD-1 and LAG-3. YY1 causes the expression of PD-1 on activated CD8^+^ T cells and their binding to the ligand PD-L1 on different cells of the TME, promoting a survival pathway instead of apoptosis [99]. Liu et al. [100] reported how the combination of PD-L1 and YY1 inhibitors can have greater effects on the cancer than either of the therapies alone. They found that YY1 can specifically bind with the de-ubiquitination enzyme USP7 [100]. USP7 can prevent YY1 from ubiquitin-dependent degradation and stabilizes YY1 expression, which can promote the proliferation, migration, and Epithelial–mesenchymal transformation (EMT) of hepatocellular carcinoma cells (HCCs). They then used Isorhamnetin (ISO) to target USP7, which in turn would then promote YY1 ubiquitin-dependent degradation and stop the overexpression of YY1. This would then promote the degradation of YY1 and result in the inhibition of EMT. They made nanoparticles that specifically targeted tumor cells and had a controlled release of ISO, for when they wanted it to release. The nanoparticles were Isorhamnetin (ISO) and anti-PD-L1 antibody dual-functional mesoporous silica nanoparticles (HMSN-ISO@ProAPD-L1 Ab), which were prepared as an anti-tumor drug to play a synergistic anti-tumor role. They then used this, and it showed a better effect than the usual PD-L1 antibody treatment. They then used a combination of PD-L1 and the nanoparticles, and this showed a promising effect on promoting T cell infiltration in these tumors [100]. This experiment shows the promising result that YY1 and PD-1 double inhibition can be a stronger treatment for tumors than either one alone. One could assume that adding a LAG-3 inhibitor into this study may even make the tumor infiltration stronger than just the two combined. The above study, along with the previous research above that, shows a crosstalk between YY1 and both PD-1 and LAG-3. Furthermore, Dulal et al. [73] also showed that a combination anti-PD-1 therapy combined with MEK inhibition (MEK inhibits MAPK pathway) produced lasting tumor regression in CT26-innoculated BALB/c mice. The MAPK pathway activates YY1 expression, and its inhibition by MEK inhibitors leads to inhibiting both PD-1 and YY1 expressions and showing stronger anti-tumor effects than seen with just the PD-1 antibody [73]. Moreover, Blake et al. [101] provide another way in which the signaling lymphocytic activation molecule family 7 (SLAMF7) may play a role in the YY1/LAG-3 immune response. SLAMF7 previously had been shown to be regulated by YY1 [101,102,103]. Blake et al. [101] showed that mice lacking the SLAMF7 showed less PD-1 on the CD8^+^ T, cells which led to an impaired ability to reach terminal exhaustion. To further research the effects that SLAMF7 was having on T cell exhaustion, Blake et al. [101] employed unbiased, neural network-based, computational clustering using FlowSOM and dimensionality reduction using tSN. The CD8 clusters showed an exhausted phenotype that had a differing expression of YY1 to that normally seen. Furthermore, a CD4 cluster was shown to have high expressions of PD-1. They further found that the T cell exhaustion clusters had high expressions of LAG-3 and PD-1, and had higher expressions of YY1. Additionally, they discovered that SLAMF7 mediated increases in the expression of YY1 [71], and is likely one of the potential mechanisms regulating T cell exhaustion. Finally, they observed that T cells cocultured with SLAMF7+ TAMs expressed multiple inhibitory receptors: LAG-3, PD-1, and 2B4 [101].

Luo et al. [104] conducted a study that determined the expression of YY1 in esophageal carcinoma (ESCA). Through using UALCAN online analysis, they found that YY1 mRNA was significantly higher in ESCA tumors than in regular cells. Furthermore, using cBioPortal, they showed that in ESCA, CD8A and CD8B cells were negatively correlated with the expression of YY1. Furthermore, they found a correlation between YY1 expression and LAG-3 expression within these ESCA cancer cells as well [104].

### 5.2. Role of LAG-3 in Immune Evasion

LAG-3 is expressed on multiple different cell types. These include CD4^+^ T cells, CD8^+^ T cells, and Treg cells. LAG-3 is required for optimal T cell regulation and homeostasis. Furthermore, persistent antigen stimulation in cancer or chronic infection leads to chronic LAG-3 expression, promoting T cell exhaustion [15]. The motif region of LAG-3, which is a glutamic acid and proline di-peptide repeat (EP), has been suggested to bind the LAG-3-associated protein (LAP), which permits LAG-3 co-localization with CD3, CD4, and/or CD8 molecules within lipid rafts [15,105]. The primary ligand of LAG-3 is MHC-Class II, which binds to a conserved, extended loop in the LAG-3 D1 domain [15]. LAG-3 and MHC class II binding contributes to the recruitment of tumor-specific CD4^+^ T cells. Then, the surrounding cells elicit a local inflammatory response dominated by Tumor Necrosis Factor (TNF), which subsequently leads to a reduction in the CD8^+^ T cell response [15,19]. However, LAG-3 has also been shown to have other ligands that can play a direct role in CD8^+^ T cell response, unlike in the MHC Class II binding. For example, Galectin-3 (Gal-3), a 31 kDa galactose-binding lectin, has been shown to bind to LAG-3, which seems to be required for the optimal inhibition of CD8^+^ T cell cytotoxic function [15,106]. Gal-3 can be expressed on different cell types, thus exerting its regulatory function on CD8^+^ T cells via multiple mechanisms [15,21]. Another ligand that has been shown to bind LAG-3 is LSECtin, which binds to the four glycosylated sites on LAG-3 and is a member of the DC-SIGN family of molecules. LSECtin has been shown to be expressed in the liver and melanoma tumor cells. This suggests a mechanism by which LAG-3 and LSECtin can regulate CD8^+^ T cells in these environments [15,107].

Grosso et al. [108] performed a study that demonstrated that there was a population of nonfunctional CD8 T cells that expressed both LAG-3 and PD-1 [51,108]. These studies were extended to human ovarian carcinoma samples, where a significant fraction of tumor antigen-specific CD8 T cells was shown to co-express LAG-3 and PD-1 [51]. These studies showed that immunotherapy in cancer likely requires the blockade of multiple inhibitory receptors, and that LAG-3 is one of those checkpoints that can make a difference in immunotherapy. During an in vitro study using human monocyte-derived dendritic cells, it was shown that LAG-3–Ig upregulated the expression of co-stimulatory molecules and increased IL-12 expression in dendritic cells [51,109]. Further data showed the LAG-3’s role in mediating an anti-tumor effect by binding to Class II MHC. The increase in IL-12 resulted in the enhanced ability of LAG-3–Ig-matured dendritic cells to mediate TH1 response, as documented by an increased production of IFN-γ by responding T cells [51]. In addition, LAG-3 was also observed to be upregulated in CD8^+^ T cells stimulated with tumor antigens [110,111]. CD8^+^ T cells in LAG-3-deficient mice exhibited significantly higher activity than those in normal mice. These data suggest that LAG-3 expression most likely has an inhibitory effect on CD8^+^ T cells. LAG-3 has been demonstrated to directly inhibit CD8^+^ T cells via signal transduction, independent of the role of MHC II and CD4^+^ T cells described above [108,111,112]. LAG-3 has also been shown to regulate other immune cells like Treg cells, NK cells, and others, but the mechanisms remain unknown [112].

Alavi et al. [113] discovered a method of inhibition through Nicotinamide of LAG-3 to promote CD8 effector T cells and reverse the negative effects of immune suppression by LAG-3 and other inhibitors. They used flow cytometry to help determine differentiation state, and it showed that on day six to seven, cultures of CD8^+^ T cells expressing LAG-3 or TIM3 had higher levels of effector memory (TEM), and terminally differentiated effector memory T cells (TEMRA) PD-1-expressing CD8^+^ T cells. Furthermore, they showed that NAM treatment downregulates TOX and induces a decrease in the expression of TIM3 and LAG-3. They figured this out by conducting a time course shown for an average across independent experiments, whereby CD3/CD28 bead stimulation until day six was either without inhibitor treatment or with inhibitor treatment, concurrent with CD3/CD28 bead stimulation [113]. This may be a promising approach to new methods that can be used to target LAG-3 in potential cancers. It has already been proven that anti PD-1 antibodies are able to halt PD-1 receptors and promote anti-tumor CD8^+^ T cell activity [104], but this is a new method in which LAG-3 itself is altered.

## 6. Bioinformatic Analyses

Next, we explored the expression of YY1, CD274 (PD-L1), and LAG-3 in pan-cancer, using normalized and batch-corrected RSEM mRNA expression data for 14 TCGA cancer types paired with their normal tissue (thyroid carcinoma (THCA), kidney renal papillary cell carcinoma (KIRP), bladder urothelial carcinoma (BLCA), liver hepatocellular carcinoma (LIHC), head and neck squamous cell carcinoma (HNSC), breast cancer (BRCA), lung adenocarcinoma (LUAD), prostate adenocarcinoma (PRAD), esophageal cancer (ESCA), kidney chromophobe (KICH), lung squamous cell carcinoma (LUSC), kidney renal clear cell carcinoma (KIRC), stomach adenocarcinoma (STAD), and colon adenocarcinoma (COAD)).

**Figure 5:**
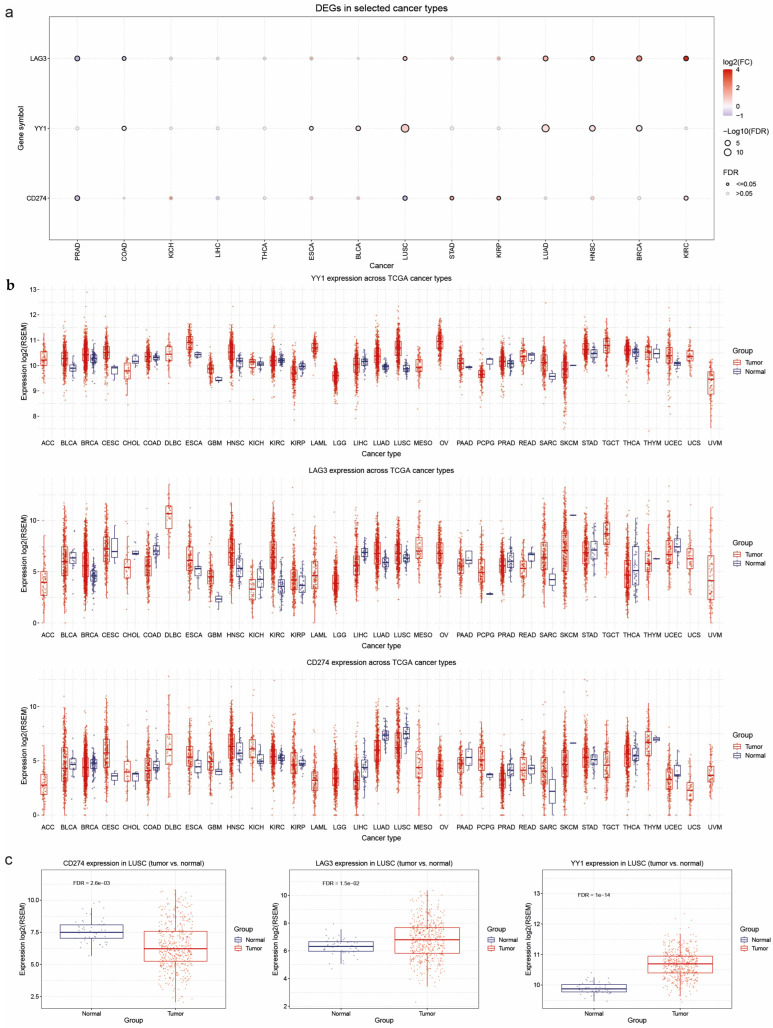
The expression of YY1, CD274 (PD-L1), and LAG-3 in pan-cancer using normalized and batch-corrected RSEM mRNA expression data for 14 TCGA cancer types paired with their normal tissue. (**a**) The bubble plot presents the fold change and FDR for gene expression across different cancer types, represented by bubble color and size. Rows indicate gene symbols, while columns correspond to selected cancer types. Bubble color transitions from purple to red, reflecting fold change (tumor vs. normal), and bubble size is proportional to FDR significance. (**b**,**c**) Boxplots display the expression levels of YY1, LAG-3, and CD274 between tumor and normal tissues across multiple cancers. A detailed example focusing on lung cancer is provided in panel (**c**), highlighting the differential expression patterns that may suggest varying roles in tumorigenesis.

Our analysis showed that LAG-3 was significantly upregulated in lung cancer (both LUAD and LUSC), HNSCC, BRCA, and KIRC, while it was significantly downregulated in PRAD. YY1 was significantly upregulated in ESCA, BLCA, LUAD, HNSCC, BRCA, and KIRC, while CD274 was upregulated in STAD, KIRC, and KIRP, and downregulated in LUSC and PRAD (Figure 5a,b). We paid specific attention to LUSC, providing exemplary analysis between paired tumor and normal tissues for each gene (Figure 5c).

**Figure 6:**
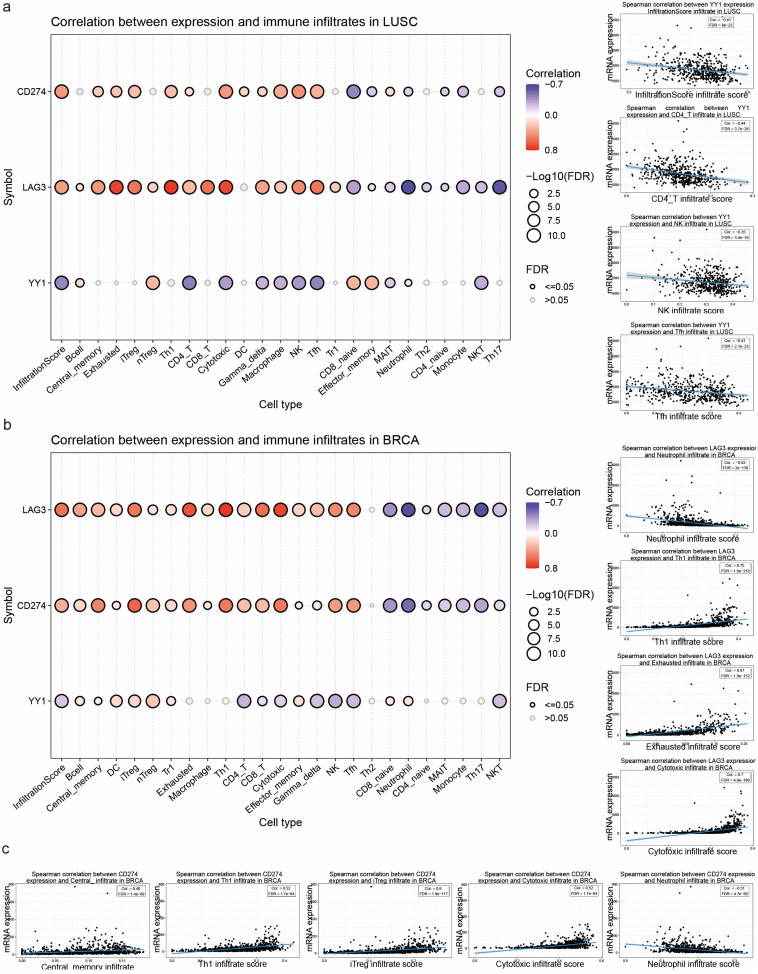
Correlation between YY1, CD274 and LAG-3 expression and immune cell infiltration in squamous cell lung carcinoma (LUSC) (**a**) and breast cancer (BRCA) (**b**). The Spearman’s test was used for correlations. The infiltrates of 24 immune cells were quantified using ImmuCellAI. Bubble size correlates with FDR significance. The black outline border indicates FDR ≤ 0.05. (**c**) Each gene’s mRNA expression was correlated with a specific immune cell’s infiltrates using scatter plots with a fitting line.

We also used ImmuCellAI to explore the correlation between YY1, CD274, and LAG-3 expression and immune infiltration in various cancers. Our bioinformatics analysis revealed many correlations between the expression of these genes and the tumor infiltration of different immune cells (Table S1). Notably, in LUSC, while LAG-3 and CD274 were positively correlated with the infiltration score (a predictor of disease prognosis) and most immune cell infiltrats, YY1 exhibited the reverse pattern, especially for CD4^+^ and CD8^+^ T cells, as well as for γδ T cells, macrophages, NK cells, Tfh, MAIT, and NKT cells (Figure 6a). A similar pattern of negative correlation was also found between LAG-3 expression and neutrophil infiltration in BRCA. On the other hand, in breast cancer, LAG-3 expression was positively correlated with the infiltration of cytotoxic and exhausted T cells, as well as with Th1 cells (Figure 6b). In addition, in breast cancer, CD274 expression was positively correlated with the infiltration of iTreg cells, cytotoxic T cells, and Th1 cells (among others), and negatively correlated with the infiltration of neutrophils in this tumor (Figure 6c).

The observed correlations between *YY1/LAG-3* expression and immune infiltration highlight their significant role in modulating the tumor microenvironment. Specifically, *LAG*-3′s positive correlation with Th1 and cytotoxic T cell infiltration suggests its involvement in driving an exhausted immune phenotype. Such exhaustion hampers anti-tumor immunity, which is a major barrier to effective immunotherapy. The bioinformatics data demonstrate that targeting *LAG*-3 could reinvigorate these exhausted T cells, potentially restoring their cytotoxic function and enabling better tumor control. Furthermore, the regulation of *LAG*-3 by *YY1* indicates that inhibiting *YY1* may concurrently suppress multiple inhibitory pathways, including *LAG*-3 and PD-1, thus enhancing therapeutic efficacy and overcoming resistance to existing checkpoint inhibitors. These findings underscore the potential of combined *YY1* and *LAG*-3 in targeting strategies to reshape the immune microenvironment, enhance anti-tumor immunity, and address the limitations of current therapies (Supplementary Table S1).

**Figure 7:**
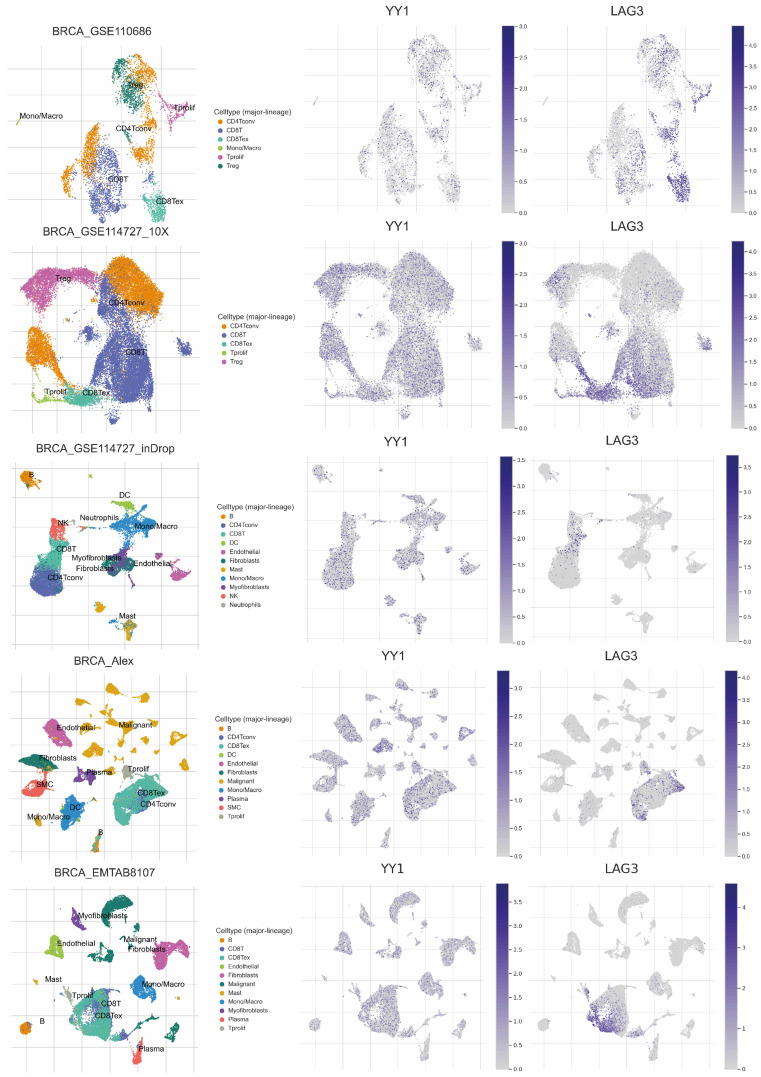
Expression of YY1 and LAG-3 in five independent scRNA-seq datasets of breast cancer (GSE110686, GSE114727_10X, GSE114727_inDrop, GSE176078 and EMTAB8107). The global-scaling normalization method (‘NormalizeData’ function) in Seurat was used to scale the raw counts (UMI) in each cell to 10,000, and to log-transform the results. YY1 and LAG-3 expression levels were calculated in log2(TPM/10+1) values and displayed using UMAP.

**Figure 8:**
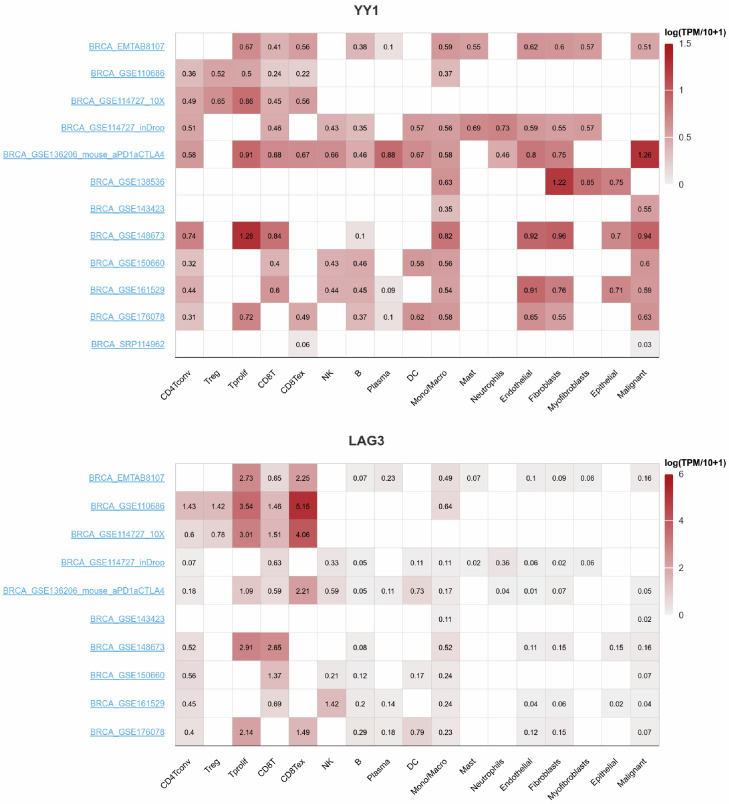
Expression of YY1 and LAG3 across different immune cells in breast cancer, using multiple GEO datasets. CD8^+^ T cells express higher levels of YY1 compared to LAG-3.

Judging from the scRNAseq data, it seems that in CD8^+^ T cells, LAG3 expression is higher compared to YY1 expression (which is lower) (Figure 7 and Figure 8). This is evident across all BRCA datasets.

### Epigenetic Regulation of LAG-3

**Figure 9:**
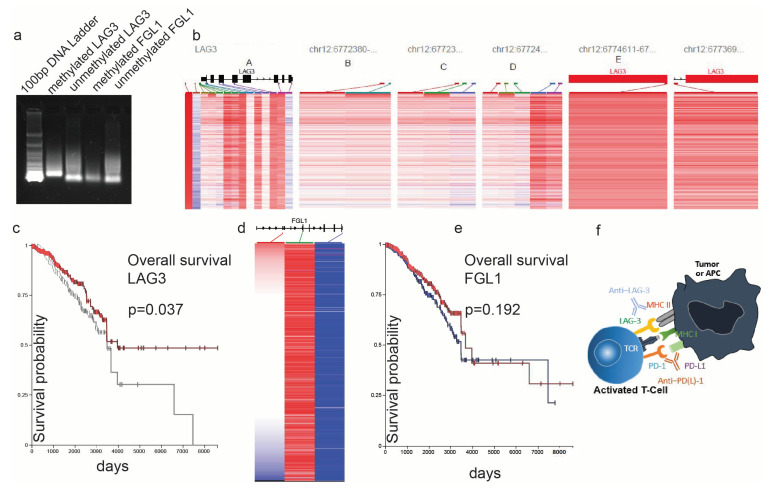
The GDC TCGA Breast Cancer (BRCA) dataset on the UCSC Xena browser was explored for LAG-3 and FGL1 methylation. (**a**) Electrophoresis result (2% agarose gel) of two methylation-specific PCR amplicons for LAG-3 and FGL1 (methylated and unmethylated DNA). (**b**) In silico analysis of LAG-3 promoter methylation using the beta values of specific markers (Illumina Human Methylation 450) shows that LAG-3 promoter is hypermethylated in breast cancer. (**c**) The Kaplan–Meier curves depict that breast cancer patients with LAG-3 hypermethylation (red curve) have better overall survival compared to those with LAG-3 hypomethylation (white curve) (*p* < 0.05, Log-rank test). (**d**) In silico analysis shows no significant methylation levels in the promoter region of FGL1. (**e**) The Kaplan–Meier curves depict no difference in the overall survival between FLG1 hyper- and hypo-methylated breast cancer patients (*p* > 0.05, Log-rank test). (**f**) Proposed model for LAG-3-expressing breast tumors (LAG-3 hyper-methylated), which could be targeted with Relatimab (anti-LAG-3) alone or in combination with anti-PD-1/PD-L1.

We set up an experimental system using the MCF-7 breast cancer cell line, and amplified LAG-3 and FGL1 after bisulfite conversion of the extracted DNA, using methylation-specific PCR (Figure 9a). We have also explored the GDC TCGA Breast Cancer (BRCA) dataset on the UCSC Xena browser, and our preliminary findings show that LAG-3 is indeed hypermethylated in its promoter region in contrast to the FGL1 promoter (Figure 9b,d). They also show that LAG-3 hypermethylation is associated with a better overall survival for breast cancer patients (*p* = 0.03, log-rank test) in contrast to FGL1 (Figure 9c,e).

## 7. Discussion

YY1 overexpression in anti-tumor CD8 T cells regulates immune evasion, in part through its transcriptional regulation of LAG-3. LAG-3 expression in CD8 T cells is activated through binding its ligands on cancer cells, resulting in the CD8 T cells being inactivated, exhausted, and therefore unable to kill the tumor cells, inducing cancer immune evasion. The anti-LAG-3 antibody is useful in inhibiting the interaction of LAG-3-expressing CD8 T cells with corresponding ligands on tumor cells, facilitating activation, and killing the tumor cells on the CD8 T cells. However, since there is more than one inhibitory receptor on CD8 T cells that might function, either independently or concomitantly, a single anti-inhibitory receptor antibody might not be sufficient to restore the activity of the CD8 T cells. There has been an early success in the combination of anti-PD-1 and anti-LAG-3 therapies [100]. However, many tumor cells are intrinsically unresponsive to checkpoint inhibitors [114]. Therefore, a different strategy to the current use of ICIs is required to overcome the ICIs’ limitations. Herein we present the potential of targeting YY1, since it directly inhibits the expression of more than one inhibitory receptor on the CD8 T cells and thereby prevents the inactivation of CD8 T cells by the ligands bearing tumor cells, preventing cancer immune evasion. In addition, YY1 also regulates PD-L1 expression in tumor cells, cell proliferation, metastasis, and resistance to therapeutics. Thus, strategies to inhibit YY1 on CD8 T cells and tumor cells have been considered as new anti-tumor immune-therapeutic modalities [11].

The general properties of YY1, including its underlying mechanisms of gene repression and activation, have been briefly discussed. Its overexpression in cancer cells is central to the pathogenesis and metastasis of many cancer types, and it has a central role in cell proliferation and resistance to cytotoxic therapeutics, including immunotherapies. YY1 is also expressed in immune T cells, with various functions on different subtypes of T cells. YY1 regulates the protein stability and gene expression of many different cancer-associated genes [1,70,71], as well as the expression of LAG-3 directly at the promoter region. It also regulates PD-1 and TIM3. YY1 expression has been shown to be transcriptionally linked to an upregulation of PD-1, TIM3, and LAG-3 [71,94], by directly binding the promoter regions [71,94]. When YY1 was knocked out, the transcription of these three gene products was decreased [71,94,95].

The recent Federal Drug Administration (FDA)’s approvals of various checkpoint inhibitors have resulted in significant clinical responses in many cancers. For example, there have been over 2000 clinical trials conducted on anti PD-1/PD-L1 according to the Cancer Research Institute. These include anti-PD-1, anti-PD-L1, and anti-PD-L2 monoclonal antibodies. However, partial responses were achieved in certain cancer types, and not all tumor types were responsive. Various mechanisms have been postulated for only the partial responses seen, such as the co-expression of other inhibitory receptors like LAG-3 and/or TIM3 that can be involved in the inactivation of CD8 T cells [71].

The expression of LAG-3 is under the control of various transcription factors that are, in part, regulated by the CD8 T cell receptors binding to the corresponding MHC-I peptide complex on the tumor cells. The expression of LAG-3 is regulated by transcriptional and post-transcriptional mechanisms that have been discussed herein. LAG-3 is required for optimal T cell regulation and homeostasis. LAG-3 binds most strongly to MHC class II, and elicits an inhibitory response to the corresponding CD4 T cells. LAG-3 binding to corresponding ligands on tumor cells promotes immune evasion in the tumor microenvironment. Therefore, the downregulation of LAG-3 can increase CD8 T cell response and reverse immune evasion.

Both YY1 and LAG-3 play a role in immune evasion by overlapping and through distinct mechanisms. In addition to YY1′s role in upregulating inhibitory receptors on T cells, it also intrinsically regulates the anti-apoptotic mechanisms in cancer cells by primarily overexpressing several anti-apoptotic gene products [1,114] and repressing immune receptors on CD8 T cells, such as Fas and DR5 [11,92]. However, LAG-3′s role in immune evasion is carried out through its activation by corresponding ligands on tumor cells and downstream inhibitory signaling pathways that inhibit cytotoxic and cytokine production by CD8 T cells. Targeting YY1 could likely lead to a decreased expression of LAG-3, PD-1, and TIM3 on CD8 T cells and, therefore, cause a lessened inhibitory response on the CD8 T cells.

The current poor clinical responses of checkpoint inhibitors in many cancers require novel approaches to inhibiting immune evasions by the inhibitory receptor–ligand interactions of CD8 T cells. YY1 targeting is an excellent candidate for inhibiting the expression of inhibitory receptors, and it has pleiotropic activities that can supersede checkpoint inhibitors. A specific inhibitor for YY1 directed at anti-tumor CD8 T cells and directed against the tumor cells will result in significant anti-tumor effects, by restoring the anti-tumor response by the CD8 T cells on the one hand, and by inhibiting the proliferation, invasion, metastases, and resistance of tumor cells to cytotoxic therapies on the other hand.

Dual targeting of YY1 and LAG-3 could potentially offer several advantages. First, YY1 simultaneously regulates multiple inhibitory receptors, including LAG-3, PD-1, and TIM-3. Therefore, this broad-spectrum inhibition can address tumor immune evasion through multiple pathways, potentially improving response rates. In addition, while existing checkpoint inhibitors focus on immune cells, YY1′s role in tumor cells—regulating PD-L1 expression, proliferation, metastasis, and resistance—provides an opportunity for dual-action therapies that target both tumor progression and immune escape mechanisms. Furthermore, evidence indicates that LAG-3 plays a role in resistance to anti-PD-1 therapies. Combining LAG-3 inhibitors with PD-1 blockade has shown promising preclinical efficacy. By including YY1 inhibitors in such combinations, one could disrupt the YY1-driven transcription of LAG-3 and PD-1, further amplifying the therapeutic benefits. Lastly, targeting LAG-3 directly addresses T cell exhaustion by promoting the effector functions of CD8^+^ T cells.

## 8. Challenges in Targeting YY1

Targeting YY1 directly on CD8 T cells or tumor cells remains the main challenge. Various approaches to targeting YY1 indirectly and systemically have been attempted, with preclinical successes [11]. However, the direct targeting of YY1 by inhibitors has not yet been very successful. Small molecule inhibitors can disrupt the interaction between YY1 and its DNA binding sites [11,100,115,116]. There is some promise in various preclinical studies; however, further investigations are needed to see the efficacy of this method. Another method for targeting YY1 was to use RNA interference. It was shown in further preclinical studies that RNA interference was able to knock down YY1, and this promoted apoptosis, inhibited cell proliferation, and enhanced the effectiveness of chemotherapy in tumor cells for these trials [11,46,116]. Moreover, the third method that has been used to target YY1 is gene editing. The use of CRISPR Cas9 has also shown some promise in preclinical studies for inhibiting YY1 [11,107,117,118]. Liu et al. [100] used ISO to help inhibit YY1. They used Isorhamnetin (ISO) to target USP7, which in turn would then promote YY1 ubiquitin-dependent degradation and stop the overexpression of YY1, as explained previously [100]. However, the direct targeting of YY1 still remains a challenge. The use of nanotechnologies may be one approach that has been successful in targeting other agents [11].

Overall, due to the pleiotropic and pro-tumorigenic activities of YY1 expression in T cells and tumor cells, and the prognostic significance of YY1 expression in cancers [1], further research is urgently needed to develop specific YY1-targeting inhibitors that will have significant anti-tumor effects on most cancers, particularly those that are resistant to immunotherapy and cytotoxic agents.

The first challenge in targeting YY1 is that since it has such a wide range of functions, inhibition can lead to unwanted responses. Although small molecule drugs have been shown to decrease the expression of YY1, they may also affect other cellular processes such as DNA replication and cell division, causing unintended consequences [11,119]. For example, in tumor cells, using a nitric oxide donor showed that the drug not only inhibited YY1 expression, but also caused cytokine release syndrome [11,120]. YY1 regulates the expression of a variety of genes, so targeting it may clearly have unintended effects on normal cells and tissues. Consequently, it is necessary to develop specific methods that can selectively target tumor cells while sparing normal cells.

Another challenge is the efficacy of YY1-targeted therapies. There have been promising results in preclinical trials; however, their efficacy in clinical settings may be limited by various factors, including drug resistance, the heterogeneity of tumor cells, and the tumor microenvironment [121]. Combining a YY1 inhibitor with an immunotherapy agent that targets the immune system’s response to tumors could enhance the immune system’s ability to recognize and destroy tumor cells, potentially improving patient survival [70]. This is one method that can be used to possibly improve the efficacy of YY1-targeted therapies.

Drug delivery to the tumor site is another challenge of targeting YY1. Developing systems to overcome this can help increase both efficacy and specificity, to help counteract some of the problems seen with YY1-specific targeting [11]. In a preclinical trial, Liu et al. [122] developed exosome-based nanoparticles that were thought to possibly enhance the efficacy of delivering YY1 inhibitors to cells. In their study, they found that in a histological analysis regarding major organs, the tissues of experimental mice were not damaged, which indicated the favorable biocompatibility of the modified exosome. They showed in their study that exosomes have great value in nucleic acid delivery and can protect therapeutic substances from degradation and clearance by the host’s immune system [122]. Nanoparticles are a strong new possible method to enhance the efficacy of YY1-targeting methods and need to be further tested in the future.

Focusing on the complex biology of YY1 and the administration of therapies that directly target YY1 could bring about the development of new and more effective treatment strategies and improve patients’ outcomes.

## 9. Conclusions

YY1 is a transcription factor that is essential to numerous cellular processes and the regulation of transcriptional and post-translational modifications of various genes. YY1 regulates various immune-associated receptors, including PD-1, LAG-3, FAS, TRAIL, and DR5. In addition, YY1 regulates PD-L1 on tumor cells and is intimately involved in the pathogenesis of tumor cells. Thus, targeting YY1 inhibition in CD8 T cells and tumor cells is a novel strategy to evade immune evasion and enhance the anti-tumor immune response, as well as to inhibit various pro-tumorigenic activities. Further studies are important to develop selective YY1-targeting agents and test them for their anti-tumor efficacies in preclinical studies, followed by clinical studies.

## Data Availability

Genomic data were extracted from TCGA (https://portal.gdc.cancer.gov/, accessed on 1 August 2024).

## Supplementary Materials

The following supporting information can be downloaded at: https://www.mdpi.com/article/10.3390/cancers17010019/s1.

## Abbreviations

Endothelial–Mesenchymal Transition (EMT), Esophageal Carcinoma (ESCA), Forkhead box protein 3 gene (Foxp3), Galectin 3 (Gal-3), Interleukin 2 (IL-2), Lymphocyte-Activation Gene 3 (LAG-3), Mitogen-activated Protein Kinase (MAPK), Mitogen-activated protein kinase (MEK), Major Histocompatibility Complex II (MHC Class II), Micro-RNAs (miRNAs), Nuclear Factor-Kappa Beta (NF-κB), Programmed Death-Ligand 1 (PD-L1), Programmed Death 1 (PD-1), Raf-1 kinase inhibitory protein (RKIP), T cell Immunoglobulin mucin-3 (Tim3), Regulatory T cells (Treg), Signaling Lymphocytic Activation Molecule Family 7 (SLAMF7), Ying Yang 1 (YY1).
